# Supplementary material for: Root fragment weight and carbohydrate dynamics of two weedy thistles Cirsium arvense (L.) Scop. and Sonchus arvensis L. during sprouting
Source: PeerJ. 2025 Apr 4;13:e19155. doi: 10.7717/peerj.19155 (PMC11974545; doi:10.7717/peerj.19155)
Supplement: Supplemental Information 2 — For temperature (C°), photoperiod (h), experimental time (growing degree days [GDD]) per week and cumulated and number of harvests. [file peerj-13-19155-s002.docx]

|  | Temperature range (C°) | |  |  | Experimental time | | |
| --- | --- | --- | --- | --- | --- | --- | --- |
| Week | Min | Max | Month | Photoperiod (h) | GDD | GDD (Cum) | Harvest |
| 1 | 5 | 12 | April | 13 | 59.5 | 59.5 |  |
| 2 | 5 | 13 |  | 13.5 | 63 | 122.5 |  |
| 3 | 6 | 14 |  | 14 | 70 | 192.5 |  |
| 4 | 7 | 15 |  | 14.5 | 77 | 269.5 | 1 |
| 5 | 8 | 16 | May | 15 | 84 | 353.5 | 2 |
| 6 | 9 | 17 |  | 15.5 | 91 | 444.5 | 3 |
| 7 | 10 | 18 |  | 16 | 98 | 542.5 | 4 |
| 8 | 11 | 19 |  | 16.5 | 105 | 647.5 | 5 |
| 9 | 12 | 20 | June | 17 | 112 | 759.5 | 6 |
| 10 | 13 | 21 |  | 17 | 119 | 878.5 | 7 |
| 11 | 14 | 22 |  | 17 | 126 | 1004.5 | 8,9 |
| 12 | 15 | 23 |  | 17 | 133 | 1137.5 | 10 |
| 13 | 16 | 24 | July | 17 | 140 | 1277.5 | 11 |
| 14 | 17 | 25 |  | 16.5 | 147 | 1424.5 | 12 |
