# Supplementary material for: Root fragment weight and carbohydrate dynamics of two weedy thistles Cirsium arvense (L.) Scop. and Sonchus arvensis L. during sprouting
Source: PeerJ. 2025 Apr 4;13:e19155. doi: 10.7717/peerj.19155 (PMC11974545; doi:10.7717/peerj.19155)
Supplement: Supplemental Information 3 — Occurring in greenhouse during first (Experiment 1) and second (Experiment 2) run of the experiment. [file peerj-13-19155-s003.docx]

|  | Global radiation (W m^-2^) | |
| --- | --- | --- |
| Months | Experiment 1 | Experiment 2 |
| April | 199.4 | 179.38 |
| May | 201.7 | 225.52 |
| June | 245.8 | 230.10 |
| July | 207.1 | 200.34 |
