# Supplementary figures and images for: Root fragment weight and carbohydrate dynamics of two weedy thistles Cirsium arvense (L.) Scop. and Sonchus arvensis L. during sprouting
Source: PeerJ. 2025 Apr 4;13:e19155. doi: 10.7717/peerj.19155 (PMC11974545; doi:10.7717/peerj.19155)

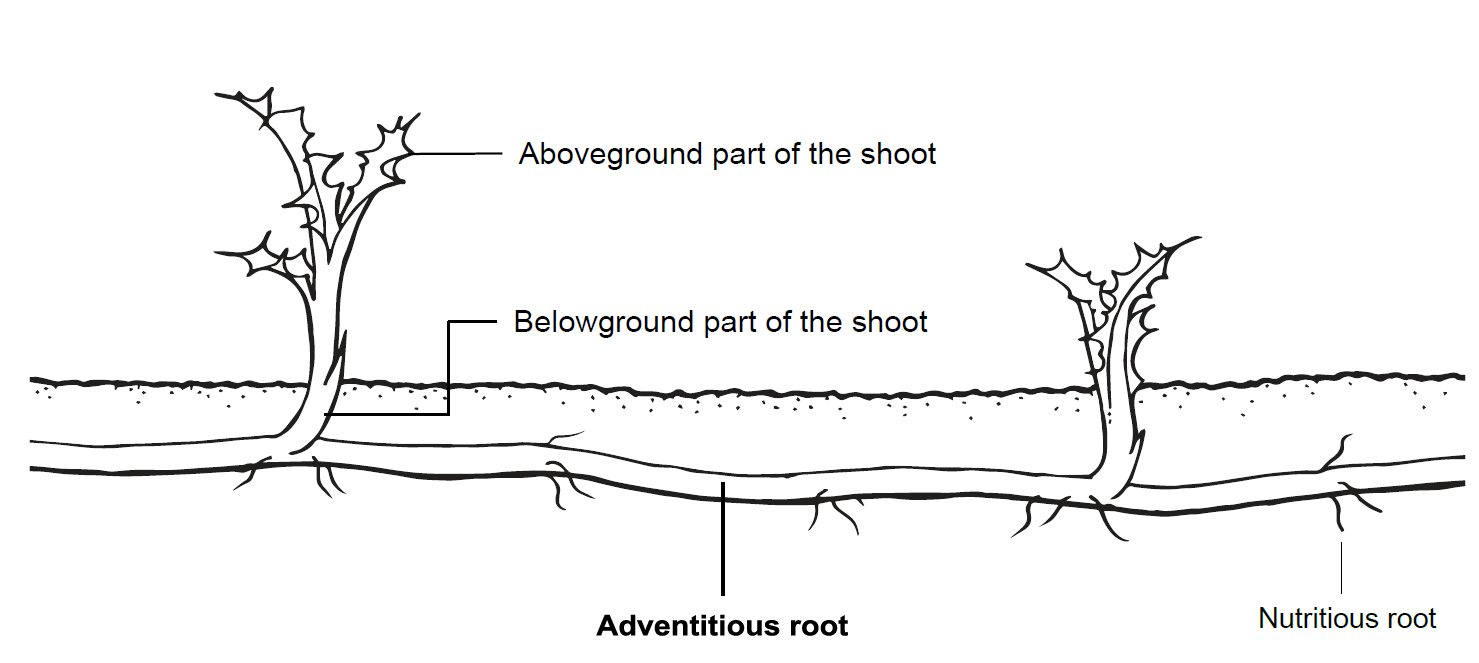

Supplement: Supplemental Information 4 — The structural differentiation between the aboveground and belowground parts of the plant. Only the adventitious roots, highlighted in bold, were used for reserve substance analysis. [file peerj-13-19155-s004.png]
